# Dkk1 inhibits malignant transformation induced by Bmi1 via the β‐catenin signaling axis in WB‐F344 oval cells

**DOI:** 10.1002/2211-5463.13132

**Published:** 2021-06-09

**Authors:** Jinjun Ye, Le Xin, Jidong Liu, Tao Tang, Xing Bao, Yukuang Yan

**Affiliations:** ^1^ Department of General Surgery Longgang Central Hospital Shenzhen China

**Keywords:** Bmi1, Dkk1, hepatocellular carcinoma, malignant transformation, WB‐F344 oval cell, β‐catenin

## Abstract

Dickkopf‐1 (Dkk1) is an inhibitor of Wnt signaling involved in cancer cell proliferation, apoptosis, and migration and angiogenesis. It was previously reported that B cell‐specific Moloney mouse leukemia virus integration site 1 (Bmi1) activates the Wnt pathway by inhibiting the expression of DKK1 in breast cancer cell lines and 293T cells. Bmi1 and DKK1 are highly expressed in liver samples taken by biopsy from patients with hepatitis B virus‐related hepatocellular carcinoma (HCC), but the effect of both Bmi1 and DKK1 on the carcinogenesis of adult hepatic stem cells (oval cells) has not previously been reported. In this study, we used WB‐F344 cells to explore the function and regulation of Dkk1 upon Bmi1 treatment. Overexpression of Dkk1 repressed differentiation, proliferation, and migration induced by Bmi1 but promoted the apoptosis of hepatic WB‐F344 oval cells. In addition, Dkk1 reduced the enhancement of β‐catenin levels induced by Bmi1. Finally, we used transcriptome sequencing to perform a comprehensive evaluation of the transcriptome‐related changes in WB‐F344 oval cells induced by Dkk1 and Bmi1. These results may provide evidence for future studies of the pathogenesis of HCC and the design of possible therapies.

## Graphical abstract

We used WB‐F344 cells to explore the function and regulation of Dickkopf‐1 (Dkk1) upon B cell‐specific Moloney mouse leukemia virus integration site 1 (Bmi1) treatment. Overexpression of Dkk1 repressed differentiation, proliferation, and migration induced by Bmi1 but promoted the apoptosis of hepatic WB‐F344 oval cells. In addition, Dkk1 reduced the enhancement of β‐catenin levels induced by Bmi1.

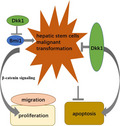

**Abbreviations**
AFP: alpha‐fetoprotein
ALB: albumin
Bmi1: B cell‐specific Moloney mouse leukemia virus integration site 1
Dkk1: Dickkopf‐1
GGT: γ‐Glutamyl transferase
GST pi: glutathione S‐transferase pi
HBV: hepatitis B virus
HCC: hepatocellular carcinoma
TGF: transforming growth factor alpha
TNF: tumor necrosis factor

Dickkopf‐1 (DKK1) was identified as a secreted protein in *Xenopus laevis*, and it acts as an inhibitor of Wnt signaling [1]. The subcellular location of the DKK1 protein is mainly the cytoplasm, and it is expressed at the highest level in the kidneys, followed by the liver and brain during the embryonic period and often in the prostate after birth [2]. Wnt signaling can be regulated by the negative feedback of DKK1 [3]. As an important regulator of Wnt signaling, DKK1 is involved in cancer cell proliferation, apoptosis, and migration and in angiogenesis [4]. Zhang *et al*. found that DKK1 plays an oncogenic role in hepatocellular carcinoma (HCC) by activating the Wnt/β‐catenin signaling pathway, which mediates the proliferation and tumorigenicity of HepG2 and HUH‐7 cells [5]. During mouse heart development, both DKK1 and DKK2 negligibly inhibit Wnt signaling in the regulation of early cardiomyocyte proliferation [6]. In addition, DKK1 is regarded as a novel marker for hepatoblastomas and intrahepatic cholangiocarcinoma [7, 8]. Furthermore, DKK1 may be a negative prognostic biomarker of HCC [9, 10]. Currently, DKK1 expression is abnormal in many kinds of tumors, and there is a significant correlation between DKK1 expression and cancer prognosis. DKK1 expression increases during the early onset of prostate cancer, and its high expression is further associated with overall shorter patient survival [11]. The increased serum DKK1 level is related to poorer recurrence‐free survival and overall survival in colorectal cancer liver oligometastases [12]. However, the expression of DKK1 and soluble frizzled‐related proteins decreased in one kind of tumor. For example, screening from the GSE15417, GSE16515, and GSE28735 pancreatic ductal adenocarcinoma and normal pancreatic tissue microarray datasets revealed that DKK1 was downregulated in pancreatic ductal adenocarcinoma compared with normal tissues. DKK1 was associated with pancreatic ductal adenocarcinoma prognosis in a Kaplan–Meier survival analysis with log‐rank test [13].

Although DKK1 is classified as a Wnt inhibitor, its role in cancer remains controversial. In some cancers, it plays a role as a tumor suppressor, while in others, such as liver cancer, breast cancer, ovarian cancer, lung cancer, and kidney cancer, it has been reported to promote tumors [14]. B cell‐specific Moloney mouse leukemia virus integration site 1 (Bmi1) is one of the members of the Polycomb family and plays an important role in the cell cycle, cell immortality, and senescence. Bmi1 was reported to regulate the proliferation of different kinds of cells, such as normal cells, stem cells, and progenitor cells [15]. It has been reported that the overexpression of Bmi1 in the WB‐F344 and OC3 cell lines promoted cell proliferation, colony formation, and invasion *in vitro* [16]. Cho *et al*. [17] found that Bmi1 activated the Wnt pathway by inhibiting the expression of DKK1 in breast cancer cell lines and 293T cells, which led to the upregulation of c‐myc, which in turn further upregulated Bmi1 through the E‐box. Bmi1 and DKK1 were highly expressed in liver samples taken by biopsy from patients with hepatitis B virus (HBV)‐related HCC, but they were not expressed in HCC samples without HBV infection [18]. There has been no report on the effect of both Bmi1 and DKK1 on the carcinogenesis of adult hepatic stem cells (oval cells).

Hepatic oval cells are immature progenitor cells in the bile duct in the liver with the capacity for self‐renewal and unrestricted proliferation. When liver damage is chronic or when the proliferation of hepatocytes is inhibited, hepatic oval cells differentiate into hepatocytes and cholangiocytes for cell replacement and organ repair in response to severe liver injury and/or compromised hepatocyte function [19]. WB‐F344 oval cells are derived from a single clone of epithelial cells isolated from rat liver and can be used as a culture analog of liver precursor cells. They have certain phenotypic and functional characteristics similar to those of hepatocytes and bile duct cells [20]. When implanted into the rat liver, WB‐F344 oval cells acquired the morphological and functional characteristics of hepatocytes. When transplanted into the liver of rats with chemical transformation, WB‐F344 oval cells developed into HCC *in vivo* [21, 22].

Herein, we report the use of rat liver oval‐like progenitor cells (WB‐F344) to investigate the function and regulation of DKK1 and Bmi1 on their cellular phenotype. The results of this study provide new insight into DKK1 and Bmi1 functions and new perspectives for designing novel therapeutic strategies for HCC.

## Materials and methods

### Cell culture and transfection

Rat hepatic oval cell line WB‐F344 was used in this study. In brief, the WB‐F344 cell line was maintained in Dulbecco's Modified Eagle Medium high glucose medium (Invitrogen, Carlsbad, CA, USA) supplemented with 10% FBS, 1% penicillin/streptomycin (10 ng·mL^−1^ penicillin and 10 U·mL^−1^ streptomycin) at 37 °C in a humidified 5% CO_2_ incubator. Control plasmid and plasmids pcDNA3.1 carry rat DKK1 and Bmi1 ORF which were procured from Origene (Rockville, MD, USA) were transfected with a lipofectamine 3000 transfection reagent (Invitrogen, Eugene, OR, USA) when the cell density was up to 60–70%.

### Reverse transcription and quantitative real‐time PCR

Reverse transcription was performed according to the manual of manufacturers (Invitrogen, Eugene). In brief, 500 ng of RNA was used for each reverse transcription reaction. Quantitative real‐time PCR was carried out with SYBR Green master mixed in Applied Biosystems 7300 Real‐time systems. Relative gene expression was calculated using 2‐ΔΔCt method. The expression of genes of interest was normalized to GAPDH. Each experiment was performed in triplicate to enhance the significance of results. Real‐time PCR analysis was carried out with the following primers: DKK1, forward: TGGAACTCCCCTGTGATTGC, reverse: CTTGCGTTCTGCACCCTAGA; Bmi1, forward: GGCTGGATGCCAAGTGGTCTT, reverse: TGAAGTACCCTCCACACAGGA; GST‐pi, forward: TTTCGCCGCCGCAGTCT, reverse: TCCACGGTCACCACCTCCTC; TNF‐a, forward: CAACAAGGAGGAGAAGTTCC, reverse: GAAGAGAACCTGGGAGTAGATAAG; GGT, forward: CATCGTGGATAAGGACGGCA, reverse: GAAGTCGGGTGTGACCTCTG; GAPDH, forward: CCATCAACGACCCCTTCATT, reverse: CACGACATACTCAGCACCAGC.

### Immunoblotting and immunofluorescence

For immunofluorescence, cells were seeded on coverslips in a 24‐well plate with a density of 8 × 10^4^ cells/well. After corresponding treatment, cells were washed with pre‐warmed PBS and fixed with 4% paraformaldehyde. The cells were permeabilized with 0.1% Triton X‐100 (Sigma Aldrich, St. Louis, MO, USA) and blocked with 5% goat serum (Sigma Aldrich), then incubated overnight with primary antibodies, including albumin (ALB) and AFP, and then, the cells were washed and incubated with Alexa 488‐conjugated secondary antibodies. Afterward, the coverslips were mounted with DAPI (Sigma Aldrich). The pictures were taken by LSM 710 (Zeiss, Germany) confocal microscope with 40× objective lens.

For apoptotic bodies—Hoechst staining, use Hoechst dyes at 1 µg·mL^−1^ and add the dye to complete culture medium. Remove culture medium from the cells and replace with medium containing dye. Incubate cells at room temperature or 37 °C for 5–15 min and then image with confocal microscope with 20× objective lens.

For immunoblotting analysis, cells in different groups were harvested after 48 h of transfection and lysed in lysis buffer (Sigma Aldrich) with 1× cocktail inhibitor (Merck, Darmstadt, Germany). Cellular protein resolved by SDS/PAGE was immunoblotted as previously described. All the primary antibodies and secondary antibodies used for tested proteins are listed in Table 1.

**Table 1:** The information of all the primary antibodies and secondary antibodies.

Name	Host	Company	ID
ALB	Rabbit	Abclonal	A0353
AFP	Rabbit	Abclonal	A0200
β‐Catenin	Rabbit	Cell Signaling Technology	8480
Cleaved‐caspase‐3	Rabbit	Abclonal	A19654
Bcl‐2	Rabbit	Abclonal	A19693
GAPDH	Rabbit	Cell Signaling Technology	5174
HRP‐conjugated anti‐rabbit IgG antibody	Goat	Cell Signaling Technology	7074S
Alexa Flour 488‐conjugated anti‐rabbit IgG antibody	Goat	Abcam	ab150077

### Cell proliferation and migration assay

For proliferation assay, cells were seeded in 96‐well plate (2000 cells per well). Hundred microliter CCK8 (Dojindo, Komamoto, Japan) reagent was added to cell culture after the plasmids were transfected for 48 h and absorbance at 450 nm was measured after 1 h of incubation. For migration assay, cells in serum‐free medium were seed in the upper chamber of Transwell insert (Corning, Corning, NY, USA) in 24‐well plate (1 × 10^5^ cells per well), with medium containing 10% FBS in the bottom chamber. After 24 h of incubation, migrated cells at the bottom side of the insert membrane were stained with crystal violet. The same procedure was used for invasion assay except that medium in the upper chamber was coated with Matrigel (BD Bioscience, Franklin Lakes, NJ, USA). At least, five random fields were photographed and counted using a phase‐contrast inverted microscope.

### mRNA extraction and RNA sequence analysis

Total RNA was extracted from the two biological repeats in each group by using TRIzol reagent (Invitrogen, Carlsbad) according to the manufacturer's instructions. The concentration and purity of RNA samples were detected by using Qubit 2.0. The integrity of total RNA was analyzed by using Bioanalyzer 2100 (Agilent, Santa Clara, CA, USA). The mRNA library was constructed according to instructions from the NEBNext® Ultra™ RNA Library Prep Kit for Illumina® (New England Biolabs, Inc., Ipswich, MA, USA). At last, PCR products were purified (AMPure XP system), and library quality was assessed on the Agilent Bioanalyzer 2100 system. The library preparations were sequenced on an Illumina Hiseq platform and 150 bp paired‐end reads were generated.

### RNA‐seq data processing

Sequencing reads were aligned to the Rat RefSeq‐RNA rn6 reference using Hisat2 v2.0.5. Differential expression analysis of two conditions/groups (two biological replicates per condition) was performed using the DESeq2 R package (1.16.1). DESeq2 provided statistical routines for determining differential expression in digital gene expression data using a model based on the negative binomial distribution. The resulting *P*‐values were adjusted using the Benjamini and Hochberg's approach for controlling the false discovery rate. Genes with an adjusted *P*‐value < 0.05 and absolute fold change of 2 found by DESeq2 were assigned as differentially expressed.

### Statistical analysis

All samples are performed for three independent measurements. Statistical analysis was presented with Student's *t*‐test to compare the different groups using graphpad prism 5.0 (GraphPad Software, La Jolla, CA, USA). Fisher's exact test was employed to filter the significant GOs and KEGG pathways using edger 3.3.1. *P* values were used to test the reliability of the analysis. The rich factor was the value of ratio between the number of differential genes enriched in the pathway and the number of annotation genes. A probability value of < 0.05 considered to be statistically significant. The level of significance was set as *P < 0.05, **P < 0.01, ***P < 0.001.

## Results

### Effects of the overexpression of Dkk1 and Bmi1 on WB‐F344 hepatic differentiation

**Fig. 1:**
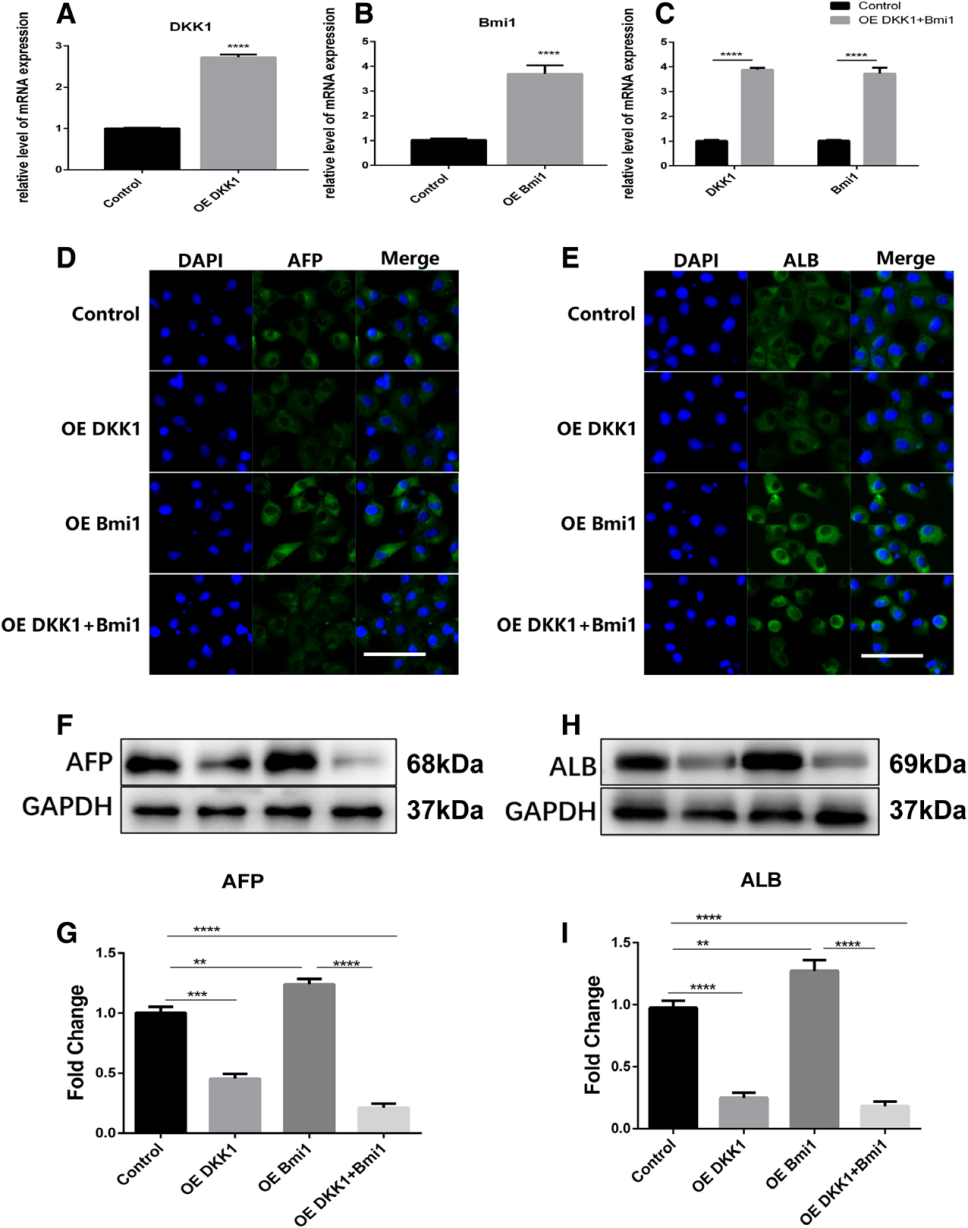
The overexpression of Dkk1 inhibited hepatic differentiation in WB‐F344 oval cells. A‐C. Dkk1, Bmi1, both Dkk1 and Bmi1 were respectively overexpressed in WB‐F344 oval cells by their plasmids. The transfection efficiency was examined with the qRT‐PCR examination. D‐I. The effect of Dkk1, Bmi1, both Dkk1 and Bmi1plasmids on the expression of hepatocyte markers in WB‐F344 oval cells were determined by immunofluorescence and immunoblotting. Data represent the mean ± SEM from at least three experiments. Student’s t‐test was performed by using GraphPad Prism 5.0. *P<0.05, **P<0.01, ***P<0.001 and ****P<0.0001. Scale bar = 50 μm.WB‐F344 oval cells control group; OE Dkk1, the Dkk1 overexpression group; OE Bmi1, t the Bmi1 overexpression group; OE Dkk1+ Bmi1, the group overexpressing both Dkk1 and Bmi1.

To determine the effects of the overexpression of Dkk1 and Bmi1 on WB‐F344 oval cells, the cells were treated with a control plasmid, Dkk1 plasmid, and Bmi1 plasmid, as well as a plasmid harboring both Dkk1 and Bmi1. The transfection efficiency was examined with qRT‐PCR and Western blot analysis (Fig. 1A–C). To determine whether the overexpression of Dkk1 and Bmi1 altered liver progenitor cell WB‐F344 hepatic differentiation, hepatocyte‐specific markers (ALB and AFP) were detected by immunofluorescence and Western blot analysis. As shown in Fig. 1D–I, the protein levels of AFP and ALB were decreased in the Dkk1 overexpression group but increased in the Bmi1 overexpression group. The protein levels of AFP and ALB were decreased in the group overexpressing both Dkk1 and Bmi1 (Fig. 1D–I). These results indicated that Dkk1 may repress WB‐F344 hepatic differentiation.

### Dkk‐1 overexpression repressed the carcinogenesis progression of WB‐F344 oval cells induced by the oncogene Bmi1 by affecting cell proliferation, migration, and apoptosis

**Fig. 2:**
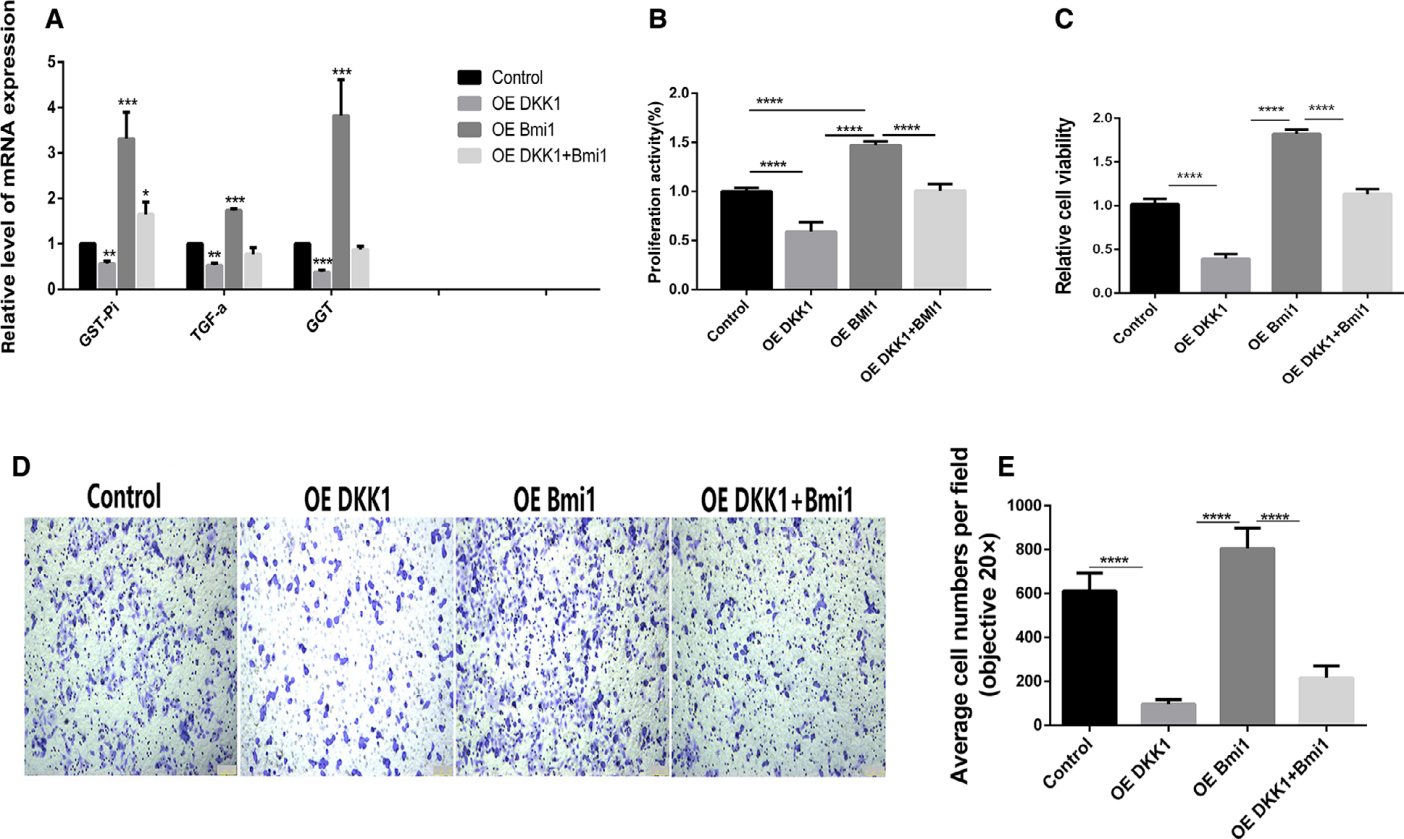
The overexpression of Dkk‐1 repressed the carcinogenesis progression of WB‐F344 oval cells induced oncogene Bmi1. A. Quantitative PCR analyses of GST pi, TGF, and GGT in WB‐F344 oval cells transfected with the plasmids of Dkk1, Bmi1, both Dkk1 and Bmi1 respectively. B‐C. Determining the cell viability and proliferation of WB‐F344 oval cells in all groups using the CCK‐8 assay. All the data were normalized by the untreated control that was set as 100%. D‐E. Cell migration was tested by transwell assays in three independent experiments. Scale bar = 100 μm. Data represent the mean ± SEM from at least three experiments, each performed with three replicates. Student’s t‐test was performed by using GraphPad Prism 5.0. *P<0.05, **P<0.01, ***P<0.001 and ****P<0.0001. Control, WB‐F344 oval cells control group; OE Dkk1, the Dkk1 overexpression group; OE Bmi1, the Bmi1 overexpression group; OE Dkk1+ Bmi1, the group overexpressing both Dkk1 and Bmi1.

**Fig. 3:**
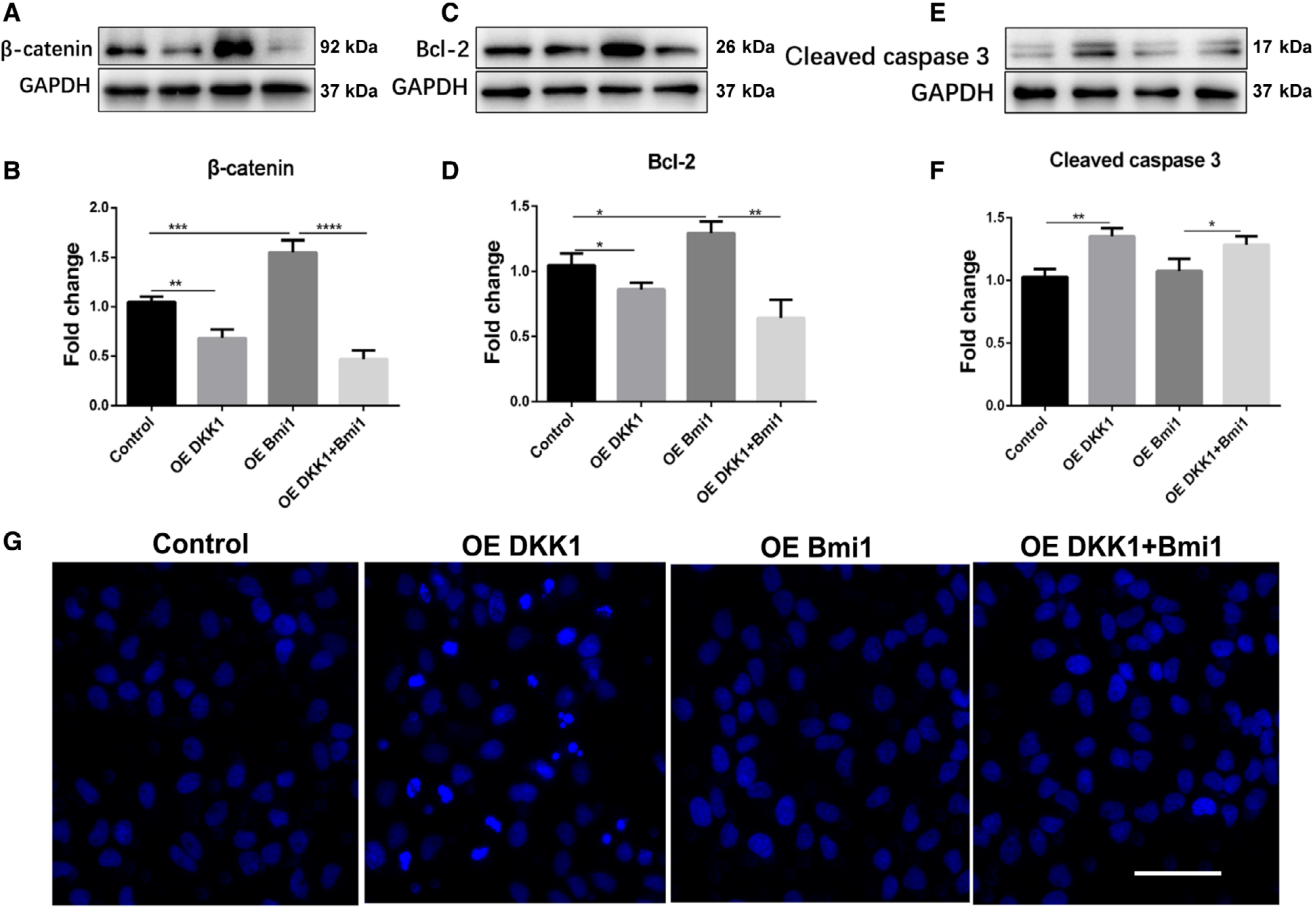
The overexpression of Dkk1 repressed the expression of β‐catenin and promoted the apoptosis in WBF344 oval cells and the role of Bmi1 contrary. A‐B. DKK1 verexpression significantly reduced the protein level of β‐catenin in in WB‐F344 oval cells. C‐F. The protein expression and quantification of apoptosis‐related markers in every group. G. DKK1 overexpression induced apoptosis in WB‐F344 oval cells. Magnification, 200X; Scale bar=50 μm. Apoptosis was assessed using a Hoechst dyes assay and confocal laser scanning microscopy was used to detect the apoptotic body. Data represent the mean ± SEM from at least three experiments. Student’s t‐test was performed by using GraphPad Prism 5.0. *P<0.05, **P<0.01, ***P<0.001 and ****P<0.0001. Control, WB‐F344 oval cells control group; OE Dkk1, the Dkk1 overexpression group; OE Bmi1, the Bmi1 overexpression group; OE Dkk1+ Bmi1, the group overexpressing both Dkk1 and Bmi1.

This study further investigated the mRNA levels of glutathione S‐transferase pi (GST pi), transforming growth factor (TGF), and γ‐Glutamyl transferase (GGT) in WB‐F344 oval cells. Significantly decreased mRNA levels of GST pi, TGF, and GGT were detected in the Dkk1 overexpression group compared with the WB‐F344 oval cells and the control plasmid group. The mRNA levels of GST pi, TGF, and GGT increased dramatically in the Bmi1 overexpression group compared with the control group. In addition, the expression of GST pi, TGF, and GGT was decreased in the group overexpressing both Dkk1 and Bmi1, suggesting that the overexpression of Dkk1 may reverse the carcinogenesis of WB‐F344 cells induced by Bmi1 overexpression (Fig. 2A). Subsequently, CCK‐8 and Transwell assays were applied to assess WB‐F344 cell viability, proliferation, and migration ability respectively. Cell viability, cell proliferation, and migration were significantly inhibited by Dkk1 overexpression. In addition, the overexpression of Bmi1 significantly induced cell proliferation and migration. However, cell proliferation and migration were inhibited by the overexpression of both Dkk1 and Bmi1 (Fig. 2B–E). Acting as a key component in the canonical Wnt signaling pathway, β‐catenin has been shown to regulate cell apoptosis. Western blot analysis was performed to measure β‐catenin and cell apoptosis‐related proteins. The experimental results suggested that cell apoptosis was positively affected by Dkk1 transfection (Fig. 3). Considering all these findings, we can conclude that the overexpression of Dkk1 repressed the progression of carcinogenic WB‐F344 oval cells induced by the oncogene Bmi1 by affecting cell proliferation, migration, and apoptosis.

### Transcriptome profiling of Dkk1 and Bmi1 overexpression separately and combined in WB‐F344 oval cells

**Fig. 4:**
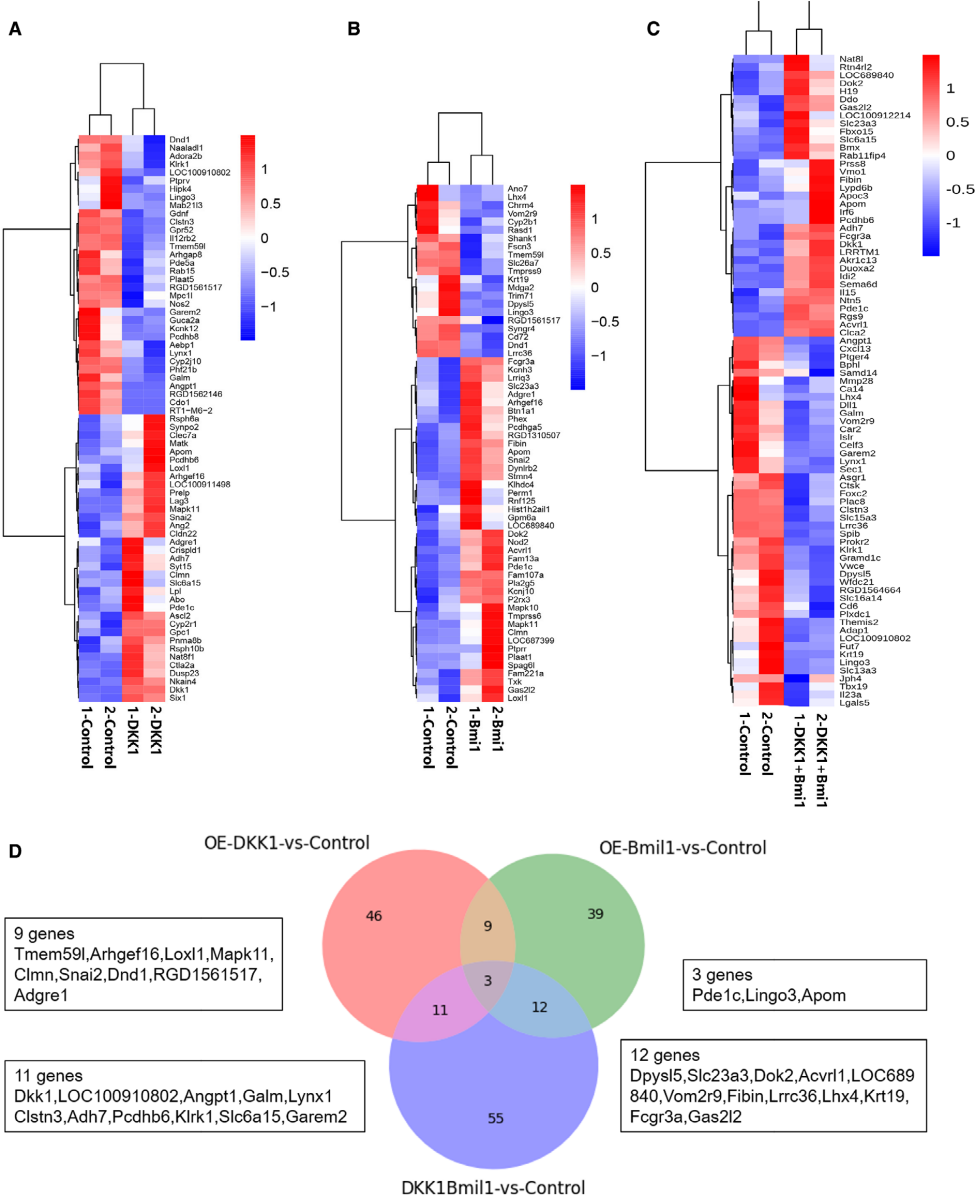
Expression profiles of mRNAs in WB‐F344 oval cells with overexpressed Dkk1, Bmi1, both Dkk1 and Bmi1, respectively. (A) Heat maps showing the differentially expressed genes in the group of overexpressed Dkk1 versus the group of control. (B) Heat maps showing the differentially expressed genes in the group of overexpressed Bmi1 versus the group of control. (C) Heat maps showing the differentially expressed genes in the group of overexpressed both Dkk1 and Bmi1 versus the group of control. The right column shows the genes. The red‐colored pixels correspond to an increased abundance of the gene in the indicated sample, whereas the green pixels indicate decreased levels (at least 2.0‐fold changes and *P* = 0.05). (D) Overview of the amount of significantly enriched genes in the different isolated groups versus the control group.

Transcriptome profiling was utilized to explore the molecular mechanisms of WB‐F344 oval cells in response to overexpressed Dkk1 and Bmi1. Sixty‐nine significantly differentially expressed genes were enriched in the overexpressed Dkk1 group and the control group (at least a 2‐fold change at *P* < 0.05). In addition, 63 differentially expressed genes were enriched in the Bmi1 overexpression group, while 81 differentially expressed genes were enriched in the group overexpressing both Dkk1 and Bmi1. A total of three genes were common among the three experimental groups, indicating a similarity between the WB‐F344‐enriched groups (Fig. 4).

### GO and KEGG pathway enrichment analysis of differentially expressed (DE) mRNAs

**Fig. 5:**
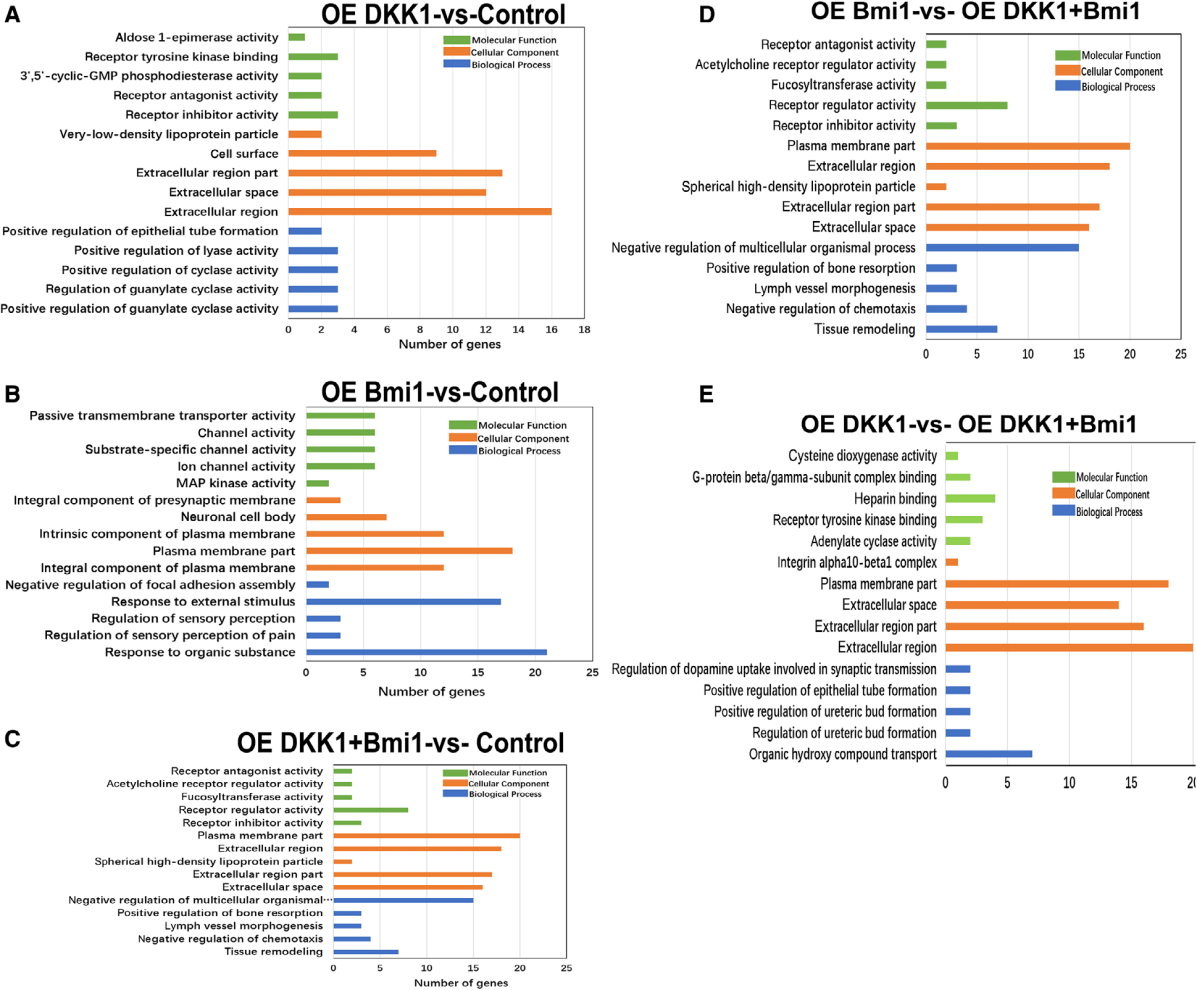
GO terms enriched by the differentially expressed genes in WB‐F344 oval cells with overexpressed Dkk1, Bmi1, both Dkk1 and Bmi1, respectively. (A) The top five most significant GO terms enriched by the differentially expressed genes in WB‐F344 oval cells with the overexpressed Dkk1. (B) The top five most significant GO terms enriched by the differentially expressed genes in WB‐F344 oval cells with the overexpressed Bmi1. (C) The top five most significant GO terms enriched by the differentially expressed genes in WB‐F344 oval cells with the overexpressed both Dkk1 and Bmi1. (D) The top five most significant GO terms enriched by the differentially expressed genes in the group overexpressing Bmi1 compared with the group overexpressing both Dkk1 and Bmi1. (E) The top five most significant GO terms enriched by the differentially expressed genes in the group overexpressing DKK1 compared with the group overexpressing both Dkk1 and Bmi1. The top five most significant GO terms were selected according to *P* value (*P* < 0.001).

Gene ontology (GO) analysis predicted the top five most significant GO terms for the significantly differentially expressed genes between the different isolated populations (Fig. 5). The analysis clearly demonstrated that some important functions were activated by both Dkk1 and Bmi1 protein overexpression. For example, receptor‐related activities include receptor inhibitor activity (Adh7, Dkk1, and Lynx1), receptor regulator activity (Adh7, Cxcl13, Dkk1, Il15, Il23a, Lynx1, Lypd6b, and Sema6d), and receptor antagonist activity (Adh7 and Dkk1). The term cellular component in the extracellular region was enriched with the most differentially expressed genes, including those in the ‘plasma membrane part’ (Acvrl1, Bmx, Car2, Cd6, Clca2, Clstn3, Dll1, Fcgr3a, Klrk1, Krt19, LRRTM1, Prokr2, Prss8, Rab11fip4, Rgs9, Rtn4rl2, Sema6d, Slc16a14, Slc6a15, and Vom2r9). Importantly, according to the GO database, we found that the enrichment terms for the group overexpressing Bmi1 compared to the group overexpressing both Dkk1 and Bmi1 revealed that the differences mainly involve the regulation of leukocyte chemotaxis (biological process), nuclear envelope lumen (cellular component), lactate transmembrane transporter activity (molecular function). The GO analysis also revealed that the biological process of the extracellular region is a very prominent feature among the 20 differentially expressed genes, such as Bche, Car2, Ccn5, Il23a, Mmp28, Ntn5, and Wnt5b) in the group overexpressing DKK1 compared to the group overexpressing both Dkk1 and Bmi1. Furthermore, organic hydroxy compound transport (biological process) and adenylate cyclase activity (molecular function) were all regulated.

**Fig. 6:**
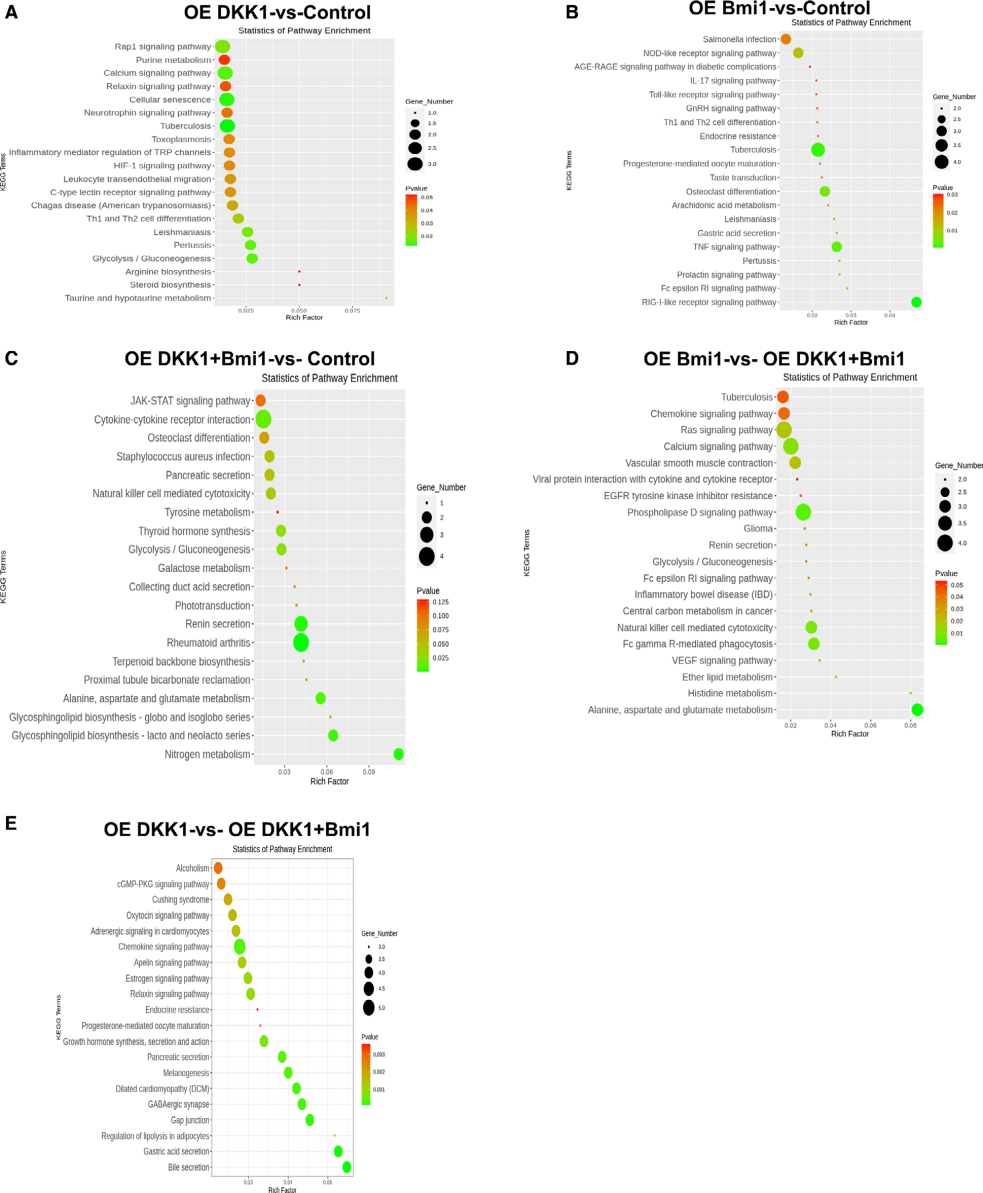
KEGG pathways enriched by the differentially expressed genes in Dkk1, Bmi1, both Dkk1‐ and Bmi1‐induced WB‐F344 oval cells, respectively. (A) Heat map of top 20 KEGG pathways enriched by Dkk1 induced genes. (B) Heat map of top 20 KEGG pathways enriched by Bmi1‐induced genes. (C) Heat map of top 20 KEGG pathways enriched by both Dkk1‐ and Bmi1‐induced genes. (D) Heat map of top 20 KEGG pathways enriched by the group overexpressing Bmi1 compared with the group overexpressing both Dkk1‐ and Bmi1‐induced genes. (E) Heat map of top 20 KEGG pathways enriched by the group overexpressing DKK1 compared with the group overexpressing both Dkk1‐ and Bmi1‐induced genes.

Moreover, this study showed that the genes in the different groups were enriched in 20 potential KEGG pathways (Fig. 6). Among these pathways, ‘Rap1 signaling pathway’ (Angpt1, Mapk11, and Adora2b), ‘cellular senescence’ (Hipk4, Mapk11, and RT1‐M6‐2), and ‘calcium signaling pathway’ (Pde1c, Nos2, and Adora2b) were the three significantly represented for the overexpressed Dkk1 group compared to the control group. The KEGG ‘RIG‐I‐like receptor signaling pathway’ (Mapk11, Rnf125, and Mapk10) and ‘TNF signaling pathway’ (Nod2, Mapk11, and Mapk10) showed enrichment with genes in the overexpressed Bmi1 group compared to the control group. In addition, genes in both the overexpressed Dkk1 and Bmi1 groups, compared with those in the control group, were significantly enriched in important subclasses, including ‘cytokine‐cytokine receptor interaction’ (Il23a, Cxcl13, Il15, and Acvrl1), ‘rheumatoid arthritis’ (Ctsk, Angpt1, Il23a, and Il15), ‘renin secretion’ (Clca2, Pde1c, and Ptger4), and ‘nitrogen metabolism’ (Car2 and Ca14). The pathway analysis of the group overexpressing Bmi1 compared with the group overexpressing both Dkk1 and Bmi1 showed that the differences are mainly in pathways involving alanine, aspartate, and glutamate metabolism and gap junctions, chemokine signaling pathways, and melanogenesis. These results imply that the genes involved in these pathways may play crucial roles in WB‐F344 cells in response to overexpressed Dkk1 and Bmi1.

## Discussion

The cause of HCC is a complex multistage process involving the expression of a large number of differential genes and resulting in the interruption of many signaling pathways. In this study, we showed that Dkk1 had a significant influence on both WB‐F344 control cells and WB‐F344 cells overexpressing Bmi1. These data may provide references to elucidate the importance of Dkk1 in HCC from a biological viewpoint. Alpha‐fetoprotein (AFP) is a hepatocyte‐specific marker [23], and ALB is the major plasma protein produced in the adult liver [24]. In our results, compared with their levels in the WB‐F344 cell control group and the Bmi1‐overexpressing group, the expression of AFP and ALB was decreased in the WB‐F344 oval cells overexpressing Dkk1, indicating that Dkk1 inhibited Bmi1‐induced differentiation of hepatic oval cells into hepatocytes. Glutathione S‐transferase pi (GST pi) is expressed in HCC and other chronic liver diseases [25]. TGF α is an important member of the epidermal growth factor (EGF) family. The level of TGF α has been found to increase in serum commensurate with the degree of liver cell injury, which may explain the role of hepatitis and cirrhosis in HCC [26]. Many studies have shown that the level of serum GGT plays an important role in the prognosis and clinicopathology of HCC [27]. In this study, the mRNA expression of GST pi and TGF α, as well as GGT, was increased with Bmi1 overexpression but decreased with Dkk1 overexpression. These results suggest that Dkk1 can reverse the malignant transformation of WB‐F344 cells induced by the overexpression of Bmi‐1. The following results of the CCK‐8 experiment and cell migration experiment also support this conclusion. In this study, the proliferation and migration activities of WB‐F344 oval cells were significantly inhibited by Dkk1 overexpression. Several studies have reported that Dkk1 is overexpressed in human HCC cell lines and elevated in HCC tissues, especially in invasive vascular tissues [28, 29]. Overexpressed Dkk1 did not influence the proliferation rate or colony formation of HepG2 cells [29]. It has been found that the high expression of Dkk1 and the accumulation of β‐catenin in the cytoplasm/nucleus of HCC are associated with poor prognosis [30]. Some experimental results have shown that Dkk1 exerts its function by promoting β‐catenin signaling [29]. In our study, the overexpression of Dkk1 promoted the apoptosis of WB‐F344 cells by reducing the expression of intracellular β‐catenin. Dkk1 plays an important role in promoting the apoptosis of tumor cells [31, 32]. Even in the absence of β‐catenin expression, Dkk1 overexpression can promote the apoptosis of H28 cells, and the apoptotic level was similar to that of H450 cells with normal β‐catenin expression [33]. Therefore, the pathogenesis of HCC may be related to the loss of the apoptosis‐promoting function of Dkk1.

Therefore, it is necessary to explore the potential molecular pathways and mechanisms of the response of WB‐F344 to overexpressed Dkk1 and Bmi1. In this study, transcriptome profiling revealed that the ‘Rap1 signaling pathway’ was regulated by the overexpression of Dkk1. Rap is one of the members of the Ras family of small GTPases. Liver pathophysiology can be regulated by rap proteins. Rap2b promotes the growth of HCC, while Rap1 may play dual roles [34]. On the one hand, the activation of the cAMP/Epac/Rap1/PI3K/Akt pathway was suggested to confer a survival effect against the apoptosis induced by Fas/bile acid. On the other hand, the cAMP/Epac/Rap1 cascade defends against TNF‐α‐induced apoptosis via a PI3K‐independent pathway [35]. Rap GEF and Epac1 activate Rap through cAMP‐binding and regulate metabolism, survival, and liver regeneration. A liver‐specific Epac2 isoform lacking the cAMP‐binding domain activates Rap1, promoting fibrosis in alcoholic liver disease. In addition, a variety of liver cell injuries can cause cell senescence, which leads to irreversible cell cycle arrest but does not affect the metabolic activity in all species. Cell senescence can prevent not only the occurrence of tumors by inhibiting the proliferation of damaged cells but can also affect the surrounding cells through the aging‐related senescence‐associated secretory phenotype [36]. Our results indicate that Dkk1 can change the cell senescence pathway in WB‐F344 oval cells. In hepatocytes, the calcium signaling pathway provides coordinates the function of the lobular metabolic region through the movement of hepatic lobules, thus supporting liver function; the transient increase in cytosolic free calcium was induced by hormones related to the formation of the second messenger inositol 1,4,5‐trisphosphate (InsP3), and its frequency increased with increasing agonist concentration. These oscillatory Ca2+ signals are thought to transmit the information encoded in the extracellular stimulus to downstream Ca2+‐sensitive metabolic processes [37]. A certain amount of Ca2+ supplementation can reverse Dkk1‐mediated Wnt/β‐catenin/canonical pathway‐related genes and proteins in primary cultured mouse osteoblasts [38].

As an oncogene, Bmi1 changed some signaling pathways related to cancer in WB‐F344 oval cells and promoted the proliferation and invasion of WB‐F344 oval cells *in vitro* [16]. The overexpression of both Dkk1 and Bmi1 was found to be related to cytokine‐cytokine receptor interactions, rheumatoid arthritis, renin secretion, and nitrogen metabolism in our study. Cytokines are broad and unbound small‐molecule proteins that are produced and released by different liver cells, and they include chemokines, interferons, interleukins, lymphokines, and tumor necrosis factor (TNF). Cytokines play important roles in inflammation and tumor progression [39]. Rheumatoid arthritis is an important outcome of the innate immune response of cells and is associated with liver injury [40]. It has been reported that the level of renin is elevated in liver cirrhosis, HCC, and hepatoblastoma [41, 42]. Moreover, this kind of increase is harmful to patients, and appropriate medical intervention measures are often used to address this increase. For many types of cancer, the nitrogen metabolism of patients is altered with detectable changes in bodily fluids, contributing to the formation of new mutations in cancer cells. The discovery of these mechanisms may promote the early diagnosis of cancer and predict the effectiveness of immunotherapy [43].

In conclusion, this study showed the functions of Dkk1 in hepatic WB‐F344 oval cell differentiation, proliferation, migration, and apoptosis. Our findings demonstrated that Dkk1 inhibited WB‐F344 oval cell migration and apoptosis induced by Bmi1 via the downregulation of the β‐catenin signaling axis. Moreover, we performed a comprehensive evaluation of the transcriptome‐related changes in WB‐F344 oval cells with the effects of overexpressed Dkk1 and Bmi1. These insights can provide certain references for the pathogenesis of liver cancer and the design of new and more reasonable therapies.

## Conflict of interest

The authors declare no conflict of interest.

## Author contributions

YY conceived and designed the project, JY acquired the data and wrote the article, LX and JL analyzed and interpreted the data, and TT and XB revised the language of the article and gave valuable opinions on the article.

## Data Availability

The data that support the findings of this study are available from the corresponding author (yanyukuang65@163.com) upon reasonable request.
